# Why Humble Farmers May in Fact Grow Bigger Potatoes: A Call for Street-Smart Decision-Making in Sport

**DOI:** 10.1186/s40798-023-00641-0

**Published:** 2023-10-14

**Authors:** Anne Hecksteden, Niklas Keller, Guangze Zhang, Tim Meyer, Thomas Hauser

**Affiliations:** 1 Chair of Sports Medicine, Institute of Sport Science, Universität Innsbruck, Innsbruck, Austria; 2 Institute of Physiology, Medical University Innsbruck, Innsbruck, Austria; 3 Simply Rational, The Decision Institute, Berlin, Germany; 4 Institute of Psychology and Ergonomics, Technical University Berlin, Berlin, Germany; 5 Harding Centre for Risk Literacy, Faculty of Health Science, University of Potsdam, Potsdam, Germany; 6 Institute of Sports and Preventive Medicine, Saarland University, Saarbrücken, Germany; 7 German Football Association, Medicine and Science, Frankfurt, Germany; 8 Faculty of Applied Sport Sciences & Personality, Business and Law School, Berlin, Germany

**Keywords:** Decision making, Heuristic, Crowd intelligence, Machine learning, Bayesian updating, Evidence, Forecasting

## Abstract

**Background:**

The main task of applied sport science is to inform decision-making in sports practice, that is, enabling practitioners to compare the expectable outcomes of different options (e.g. training programs).

**Main Body:**

The “evidence” provided may range from group averages to multivariable prediction models. By contrast, many decisions are still largely based on the subjective, experience-based judgement of athletes and coaches. While for the research scientist this may seem “unscientific” and even “irrational”, it is important to realize the different perspectives: science values novelty, universal validity, methodological rigor, and contributions towards long-term advancement. Practitioners are judged by the performance outcomes of contemporary, specific athletes. This makes out-of-sample predictive accuracy and robustness decisive requirements for useful decision support. At this point, researchers must concede that under the framework conditions of sport (small samples, multifactorial outcomes etc.) near certainty is unattainable, even with cutting-edge methods that might theoretically enable near-perfect accuracy. Rather, the sport ecosystem favors simpler rules, learning by experience, human judgement, and integration across different sources of knowledge. In other words, the focus of practitioners on experience and human judgement, complemented—but not superseded—by scientific evidence is probably street-smart after all. A major downside of this human-driven approach is the lack of science-grade evaluation and transparency. However, methods are available to merge the assets of data- and human-driven strategies and mitigate biases.

**Short Conclusion:**

This work presents the challenges of learning, forecasting and decision-making in sport as well as specific opportunities for turning the prevailing “evidence vs. eminence” contrast into a synergy.

## Key Points

- Generally, decision making in sports practice is based on “evidence “ from scientific research or on “eminence” based on experience and subjective human judgment.
- Under the framework conditions of sport—specifically complexity and sparse data—both come with limitations that lead to considerable uncertainty when making decisions in practice.
- A broader range of strategies, smart strategy combinations and critical evaluation of performance under real-world conditions contribute to better decisions and better outcomes in the field of sport and exercise.

## Background

### How to Make Good Decisions in Sports

Applied sport science aims to provide the basis for informed decisions in sports practice—and thereby for the effectiveness and safety of exercise training on all levels. While this statement may seem self-evident, framing applied sport science in the context of decision-making (providing guidance for a concrete, new case) rather than inference (using empirical data to gain new insights into the workings of nature) has important consequences. Perhaps most fundamentally, quality criteria for the output shift from the habitual set around novelty, universal validity, and contribution towards the long-term advancement of science, to a more instrumental set around helpfulness towards achieving current goals and validity under the framework conditions of the specific use case.

**Fig. 1:**
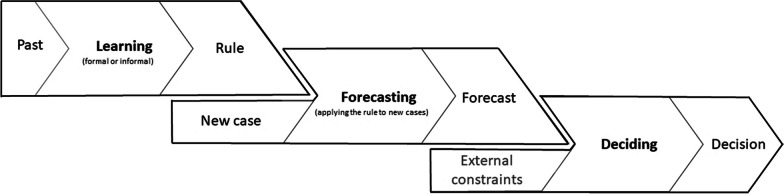
Learning, forecasting and decision making. Applied sports science contributes important groundwork for informed decision-making in sports practice—not less and not more

Figure 1 illustrates how rules learned from past observations are subsequently brought to bear in forecasting and decision-making. Reliably deciding for the best or at least for a better-than-random choice of several options (e.g. different training intensities, recovery strategies, or time points of return to sport), calls for an—at least implicit—prediction of what would happen with each of the options. This outcome-oriented perspective of the practitioner makes out-of-sample predictive accuracy a critical requirement for useful scientific evidence. Importantly, agreement with the past (e.g. previously collected data; scientists' habitual “hindsight” perspective) is a poor indicator of generalizability to new cases (practitioners' “foresight” perspective) [1]. Negligible out-of-sample predictive performance can occur despite a near-perfect agreement with the observations from which the rule has been learned [1, 2].

### Prediction Models and Uncertainty in Sports

As sport-related outcomes are usually multifactorially influenced, for a contemporary life scientist, the natural approach to the challenge of “foresight” is arguably a data-driven prediction model based on (multiple-) regression or a more fancy machine learning algorithm. In fact, a comprehensive model could theoretically transform rules previously inferred from data into accurate forecasts—thereby directly linking research and practice. An illustrative example is the field of astronomy, in which physical laws are used to calculate the future positions of celestial bodies with great precision. In the field of sport, however, such a near-clairvoyant model is not only unavailable to date but also unrealistic (given the bewildering complexity of exercise training effects and limited sample sizes [3, 4]), as well as impractical (considering the testing burden associated with assessing the multitude of input variables).

While uncertainty and ambiguity are generally unfavourable in decision making especially if stakes are high, actively embracing the limits of one's knowledge and forecasting tools is an important factor in making rational decisions [5]. An obvious advantage of such “intellectual” [5] or “epistemic” [6] humility is risk and contingency management: being aware of perhaps being wrong helps mitigate the effects of actual errors (by being attentive and prepared). A less well-recognized benefit is increased freedom in strategy selection and combination: being aware that “all models are wrong” (even the sophisticated ones currently considered as reference-standard) opens the competition for a greater variety of epistemic approaches [6].

In fact, under real-world constraints (rules inferred from small samples, unknown moderators, measurement error in assessing the specific case etc.) standardized practice based on expected values for a larger reference class can offer superior performance compared to “individualized” decisions based on a multivariable prediction model [7]. Admittedly, deliberately ignoring established influencing factors is counterintuitive. It may be even more surprising that improving predictive performance by limiting model complexity is a matter of course in bioinformatics and machine learning—uniquely “data-driven” fields that are generally thought to seek salvation in complexity and dimensionality [8]. This less-is-more effect can be traced back to the trade-off between tightly fitting the model to the training data on the one hand and the generalizability of the trained model to other samples and new cases on the other: A highly complex, flexible model will fit limited training data almost perfectly (remember from high school that two points can always be fit perfectly with a straight line, three points with a second degree polynomial etc). However, such a close fit to the training data (low bias) is achieved by fitting not only the regularities but also spurious variations leading to marked differences between models trained with different datasets (high variance). However, using a model (or rule) for making practically-useful forecasts requires its applicability (“generalizability”) beyond the cases from which it has been learned (Fig. 1). The spuriously high, “useless” performance on the training data is known as “overfitting”. Simple models generally provide more robust results (low variance) but may fail to capture as much of the regularities as possible (high bias, underfitting). Optimizing out-of-sample predictive performance requires finding the sweet spot of this bias-variance trade-off—the location of which is heavily dependent on the amount of available training data. An intuitive illustration can be found in [8]. It is important to note that overfitting is not confined to machine learning but may be seen as a special case of the “narrative fallacy”: In hindsight, we will generally find a compelling story of why and how things developed as they did. Projecting this narrative into the future is a completely different story. At this point, it is important to note that use of the terms “bias” and “variance” differs between machine learning, statistics and psychology contexts. The above paragraph reflects the machine learning perspective.

### Ecological Rationality—Generalizing the Bias-Variance Lesson

Selecting the option with the most favourable expected outcome after carefully weighing all available criteria (“maximizing expected utility”) has long been considered the essence of rational decision-making. In practice though, the limited amounts of knowledge, information, time, and computational power that human beings possess do not allow for such perfection [9]. The more benign version of the argument is the “effort-accuracy trade-off”: Searching additional information or using additional computation power improves performance but with diminishing returns—and humans stop optimizing when the additional benefit is simply not worth it. The bias-variance trade-off, however, puts this in a very different perspective: In a world marked by changes and uncertainties, additional effort (that is: more information and complex computation) can actually reduce forecasting accuracy! Put more generally, the real-world performance of a forecasting or decision-making strategy (and therefore the rational choice between strategies) depends on its fit with the framework conditions of the specific use case rather than on the strategy’s theoretical performance potential under ideal conditions. A more comprehensive exposé has been published by Gigerenzer et al. [10]. Table 1 summarizes the key ideas of ecological rationality.

**Table 1:** Ecological rationality—key ideas

**Don’t let the perfect be the enemy of the good!** Renouncing an approach that would be able to provide perfect results under ideal conditions for a simpler alternative can improve performance under real-world circumstances	
**There is no golden bullet!** The optimal strategy cannot be identified in general but depends on preconditions of the specific use case	
**The diversity of epistemic approaches is an asset** The above insights can be generalized beyond tailoring machine learning algorithms to a broader range of forecasting and decision making strategies. Examples for such alternatives range from expert intuition (“gut feeling” [11]) and deliberately simple decision rules (fast-and-frugal heuristics [9]), to debiasing subjective judgements (“crowd intelligence” [10, 12]) and the strategic integration across several sources of information and knowledge	

## The Sport Ecosystem

### Key Aspects of Ecology

The prediction ecology of a specific use case primarily concerns the framework conditions for learning rules that can subsequently be used to make useful predictions for new cases. In a wider sense, the set of options between which to decide, as well as practical constraints and evaluation criteria regarding the decision-making process and the decision maker are also part of the decision ecology. A clear and concise overview is complicated by varying terminologies and focal points associated with different approaches and the fields within which they have been developed. Here we aim to provide a unified overview by identifying four main aspects: predictability, explanatory (cues), criterion variables, and the number of precedents to learn from.

It should be noted that that when referring to the “sport ecosystem”, we take the athlete centric perspective prevailing in training science and sports medicine; i.e. making decisions in the context of optimizing the training process for specific athletes. Arguably, other sport related fields, e.g. sport economy, sport sociology or betting, have markedly different decision ecologies which are beyond the scope of this manuscript. Moreover, while many aspects characterize the sport ecosystem in general, others only apply in high performance environments.

#### Predictability

Predictability of a specific outcome can be limited by random chance playing a role in it (aleatoric uncertainty) and/or by practical epistemic constraints (epistemic uncertainty). An example of high aleatoric uncertainty is contact injuries in team sports which are due to the player being “in the wrong place and situation at the wrong time” with few (if any) risk factors that could—even in principle—be identified in advance [2]. By contrast, overload injuries are an example of mainly epistemic uncertainty: The loading capacities of e.g. a runner's metatarsal bones and the exercise-induced (local) load are existing quantities and knowable in principle, but we cannot assess or estimate them precisely. Importantly, predictability is sensitive to the timeframe. In many situations, short-term “anticipation” is easier and more accurate than long-term forecasting. A theory-based idea of predictability for the outcome in question is crucial for topic and strategy selection, risk management, and spotting exaggerated claims of model performance and overconfidence in subjective judgements. It is noteworthy that data driven approaches to estimating predictability do exist and may be of value for some sport related outcomes (e.g. goal scoring in football [13] or match outcome [14]). However, caution is necessary as this involves strong assumptions, requires huge sample sizes and entails regress issues (specifically with regard to the kind of probability distribution [15]) the relevance of which is hard-to-fathom in the specific use case.

In sport, predictability varies widely between negligible (contact injuries in team sports) and good (change in running performance with change in body weight [16, 17]). Many outcomes fall in between: they are predictable to some (potentially meaningful) extent and gradual improvements rely on identifying dominant features and smart strategy selection/combination.

**Fig. 2:**
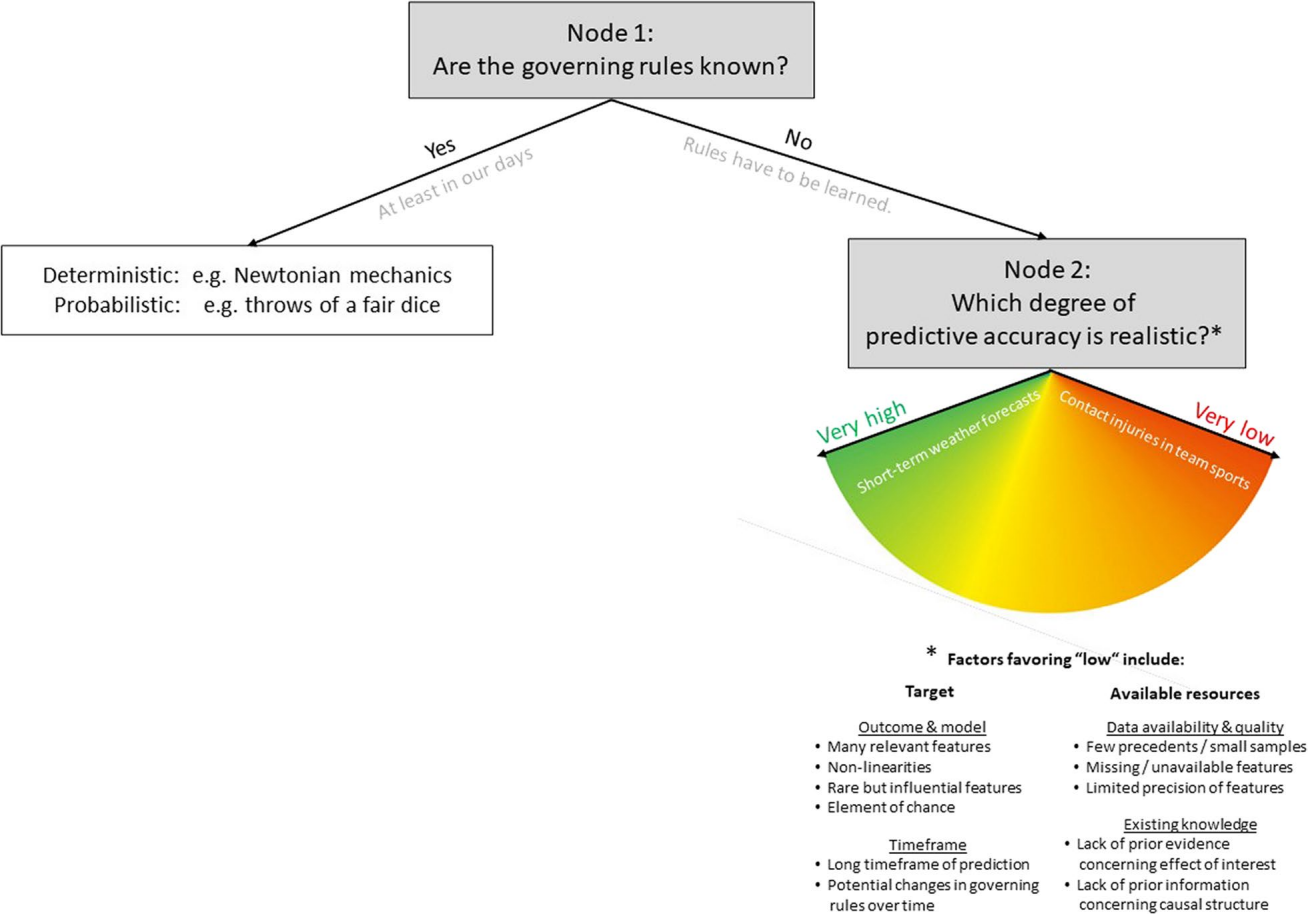
Predictability in different “worlds”. Can we realistically expect to procure generalizable rules? Note: The classification of a specific case may change over time at both nodes. Example for node 1: The laws of Newtonian mechanics once had to be discovered, but have since acquired the status of “law of nature”. Example for node 2: Conceptual and technological advances can gradually improve predictability e.g. in weather forecasting

It is important to note that there are several classifications of “worlds” tightly linked to predictability [18–22]. While the fields of origin and the dimensions differ, a unifying rationale is tentatively illustrated by the small tree in Fig. 2. There are only two nodes: (1) Are the governing rules known or do they have to be learned from limited amounts of previous observations? And (2) Can we realistically hope to learn rules with near-perfect out-of-sample predictive accuracy under real-world conditions? The world of sport is located in the lower right, reflecting limited predictability and considerable uncertainty.

#### Cues

Using currently available cues (explanatory variables) to predict the state of a complex system at a future time point is the essence of forecasting—and thereby of evaluating different options between which to decide (Fig. 1). The *number* of cues, their relative importance and potential interactions determine the complexity of the rule to be learned, and thereby the number of precedents needed to do so. The *type* of relevant cues (manifest/objective quantity, latent/complex construct, subjective perception, social) is another important aspect for matching a forecasting strategy to the environmental structure. Further aspects of the set of cues are their *redundancy* (overlap in meaning), *accessibility* (e.g. testing burden), *availability* (at the time point of making the forecast), and finally the *uncertainty of cue values* (e.g. due to measurement error).

The general, cue-related specifics of the sport ecosystem mainly concern number and type. Most outcomes of major interest in sports are multifactorially influenced with at least a considerable subset of influencing factors being of noteworthy and consistent importance. Although it is important not to confound “cue” with “cause”, a multitude of relevant explanatory factors is highly plausible. Moreover, many conceptually important explanatory factors are not manifest, directly observable quantities (e.g. the speed or the number of sprints) but complex constructs (e.g. recovery needs or movement quality) that have to be inferred from indicators of their various dimensions. Taking recovery needs as an example, indicators may include blood born markers, heart rate and heart rate variability measures and tests of neuromuscular performance [23]. While this approach is objective and scaleable, it further increases the number of parameters to be fit and thereby the sample size required for learning. As a complementary asset of the sport ecosystem, the corporeality of exercise regularly enables direct access to the target construct as a subjective perception. The human brain evolved to integrate exercise-related cues into a feeling (athlete) or impression (coach) of fatigue, movement quality etc. Again taking recovery needs as an example, questionnaire results almost uniformly outperform objective indicators—at least in the context of scientific studies with no conflicts of interest on the side of athletes or coaches [23].

#### Criterion

To develop and evaluate forecasting accuracy, it is crucial to verify agreement between predictions and actual outcomes (“ground truth” or criterion). Therefore, the timely availability of an unambiguous criterion is an important aspect of a learning environment. In machine learning this consideration is embodied in the concept of “supervised learning”, but it is equally important for (human) learning from experience. The above considerations regarding manifest, directly observable quantities and complex, latent constructs also apply to the criterion variable.

Of note, this section refers only to evaluating the forecast (e.g. an estimate of injury risk for a specific athlete and timeframe) by systematically checking its agreement with what (later) actually happens (e.g. an injury occurs or not). Importantly, this does not coincide with evaluating the decision which also has to take other factors into account and therefore requires other criteria (e.g. long term performance development or competitive success).

#### Precedents

The number of similar cases from which to learn is fundamental for obtaining generalizable rules. This consideration is embodied by the statistical proverb “Repetition is the key to separate trait from chance.” While in the life science context, the critical quantity is the number of cases or events, the rule also applies to learning by experience. Importantly, increasing the number of explanatory factors cannot compensate for a limited number of precedents but aggravates the risk of overfitting.

A characteristic feature of high-level sport is the small number of athletes [3] and even outside high-performance environments sample sizes in sport science are usually limited. Therefore, “greedy” approaches (high-dimensional biomarkers / “omics”, “deep learning”) usually fail to provide useful out-of-sample predictive accuracy [3]. It is important to note that experienced coaches may have had more opportunities for learning than any scientific trial.

### The Sport Ecosystem—Specific Aspects

Beyond the peculiar expression of the 4 general characteristics above, the sport ecosystem is characterized by sport specific aspects, 3 of which will be discussed below.

#### The Ecosystem in High-Performance Sport is Populated by Outliers

High-performance athletes are, more-or-less by definition, exceptions to the rule. This impedes the generalizability of learning outcomes and complicates the identification of implausible results.

#### Specific Constraints on Decision-Making in High-Performance Sport

Decision making in high-performance sport faces additional, specific constraints. For example, in team sports a specific number of players has to be lined up for a competitive match—even if all players have a high predicted probability of getting injured. Moreover, the trade-off between expected consequences for individual health and team success, respectively, varies between players. These aspects differ from decision-making in other areas of health care where decisions are based exclusively on the expected (health) consequences for the concerned individual [2]. Another example is the acceptability of repeated or extensive testing which might affect tightly structured training and recovery routines.

#### Importance of Avoiding the Big Mistakes

Athletic training and performance development are long-term and therefore involve a large number of decisions ranging from strategic to mundane. In this context, it is important to keep in mind that a single big mistake may outweigh the positive effects of a large number of gradual optimizations. Therefore, it is crucial to identify forecasts that are “way off” or cases that are “off limits” and for which an otherwise successful model may not be applicable (e.g. due to rare but influential characteristics [15]). Moreover, as for predictability in general, it is important to have a theory-based expectation regarding the distribution of forecasting errors. It makes a big difference for risk management whether errors are more or less normally distributed or if long streaks of accurate forecasts are punctuated by complete failures [15]. Unfortunately, infrequent but massive errors are not well represented by common measures of predictive accuracy. Therefore, plausibility checks are essential for robust decision-making with imperfect knowledge. Importantly, this requires cross-comparison between different sources of knowledge—especially when “plausible” may not be approximated by “within a group-based reference range”. To date, such non-algorithmic critical thinking and common sense are still a privilege of humans.

### The Sport Ecosystem in a Nutshell

Taken together, the key challenge of the sport ecosystem is complexity complicated by a small, sometimes tiny number of precedents to learn from. On the assets side, there is the corporeality of physical exercise which provides direct subjective access to complex exercise-related features, the feedback provided by daily practice and competition performance, and the longstanding, immersive experience of professional coaches, support staff and athletes. From the perspective of task analysis, the priority of avoiding big mistakes (robustness) and sport-specific external constraints on decision-making have to be taken into account.

## Decision-Making Strategies in Sport

**Fig. 3:**
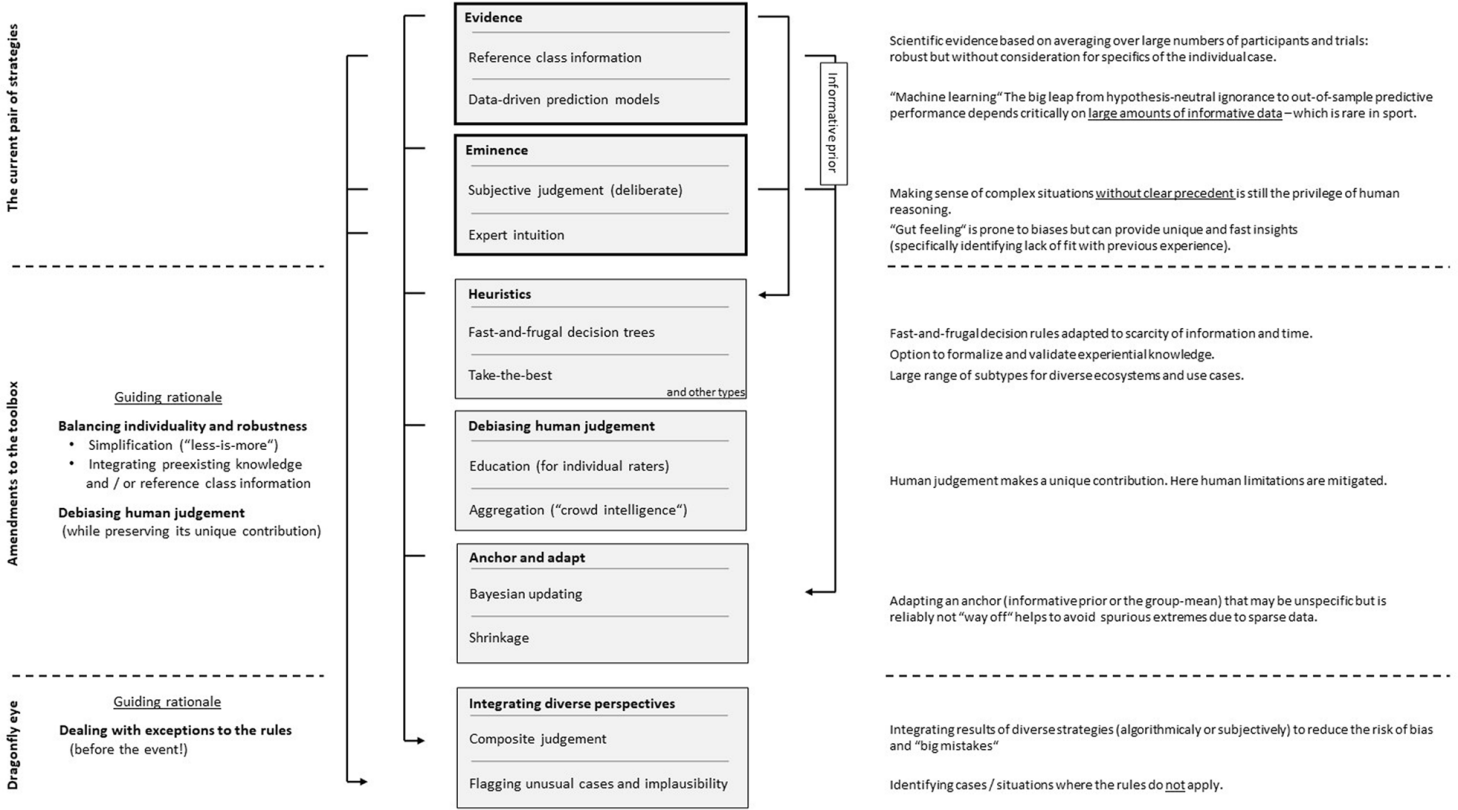
Strategy selection and combination in the sport ecosystem

In the context of sports, only two contrasting strategies are generally considered: (1) The life science approach including group-based scientific evidence and data-driven prediction models and (2) Experiential knowledge and expert intuition. However, this “evidence vs. eminence” dichotomy ignores the diversity of available options. In particular, there are two potential amendments with promising fit to the sports ecosystem: deliberately frugal decision rules (heuristics) and debiasing and aggregating subjective human judgements (“crowd intelligence”). Finally, the individual strategies are not mutually exclusive but may be synergistically combined. The following sections discuss assets, drawbacks and potential synergies. A conceptual overview is provided in Fig. 3.

### The Life Science Approach (“Evidence”)

In many ecosystems, standardized expectations based on a large reference group *(*e.g. results from large randomized controlled trials or meta-analyses) are superior to expert judgements [24] and a hard-to-beat benchmark for data-driven, individualized predictions [7]. However, it has to be kept in mind that the predictive value of trials with sample sizes typical for sport science is low [3, 25]. This means that many (perhaps most [26]) novel findings are false. While these busts will eventually be sorted out during the research process [27], they make early adoption a hazardous business for practitioners.

Data-driven prediction models aim to reduce uncertainty by considering a comprehensive set of explanatory variables. They combine the theoretical potential for.

(near-) perfect predictive performance with objectivity and scalability (e.g. by implementing the trained model in a digital decision support system). Moreover, computation capacity and large amounts of data (from wearables, player tracking, power meters, smartphone apps etc.) are readily available today. However, critical requirements for unleashing the potential of data-driven prediction models are a large number of precedents and an informative (!) panel of explanatory variables. As already pointed out, these requirements are generally not met by the sport ecosystem. Importantly, this does not rule out that in some sport-related applications data-driven prediction models may be helpful—particularly when a limited number of dominant cues or patterns can be identified [2] and/or as part of composite strategies [3, 28]. A recent illustrative example for the latter is the combination of data-driven prediction with coaches’ subjective judgment in talent identification [11].

Beyond predictive accuracy, interpretability is an important asset in the context of decision support. While in theory “black box” predictions of a relevant target (e.g. injury risk) made with a well-validated model can be useful, ideally, predictive accuracy coincides with causal interpretability. In other words, the explanatory variables relate to determinants in causal concepts and it is known which cue values drive a specific prediction. An illustrative example is the monitoring of injury risk: While an accurate estimate of injury risk may in itself be worthwhile (e.g. to avoid exposure when the risk estimate is high), knowing the factors that lead to an elevated risk would enable a more targeted response.

Finally, it has to be kept in mind that although current software packages make “machine learning” doable for the subject matter scientist, this ease is deceiving and a lack of expertise (or rigor) in the finer details of model fitting and validation can easily lead to spuriously high (!) estimates of model performance [2]. A salient but regularly overlocked pitfall is information leakage [29], the risk of which is particularly high when working with longitudinal (e.g. monitoring) data [2].

### Learning by Experience and Expert Judgement (“Eminence”)

Arguably, experience-based subjective judgments are still the prevailing basis of decision-making in sports. While from the scientist’s perspective this may sometimes seem “unscientific”, “irrational” or even stubborn, in fact, characteristic features of the sport ecosystem favour this approach. To begin with, experienced professional coaches and other support staff typically have access to more precedents than can be included in any scientific trials. Together with the direct subjective perceptibility of complex exercise-related cues, regular feedback provided during daily practice and competition, and guidance from formal training, this favours the build-up of robust experiential knowledge. Moreover, making viable forecasts in complex situations with very few clearly identifiable precedents is a characteristic feature of human reasoning. This human faculty exploits higher-order mental capacities such as thinking in analogies to make sense of diverse and incomplete information and remains hard to emulate for artificial intelligence.

It is important to note that subjective assessments do not necessarily arise from an unconscious “black box”. While this is a characteristic feature of intuitions and “gut feelings”, subjective judgements and forecasts can be the result of targeted information search and conscious reasoning with an explicit line of argument as well as an estimate of uncertainty [30, 31]. While the assets of the latter are well supported [30, 31] (particularly when complexity is combined with sparse data, as is the case in sport) the potential contribution of expert intuition and “gut feelings” is less clear. Arguably, expert intuitions should be particularly considered for spotting abnormalities e.g. cases that do not belong to the reference class despite fulfilling formal inclusion criteria [32] (Fig. 3). However, this remains to be empirically verified in the context of sport.

### Deliberately Simple Decision Rules (Heuristics)

Heuristics are simple “rules of thumb” that enable fast decisions without the effort and resources needed for considering all available information. Generally, heuristics are viewed in light of an effort-accuracy trade-off: “quick-and-dirty” solutions, necessary to get by with the deluge of everyday decisions that do not merit the effort of optimization. However, as already pointed out, simple rules can also be more accurate than extensive strategies that consider more cues and use more extensive computation methods [9]. Empirical results supporting a “less-is-more” effect have been reported in a wide range of fields [7, 33–40].

A structured introduction to the science of heuristics is beyond the scope of this work and has been provided by experts in the field [9, 41]. However, it is important to identify two main perspectives: In the seminal work of Tverski and Kahneman [42], heuristics are simplifications of judgemental operations that are unconsciously used by humans and rely on subjective cues such as representativeness (how much the specific case evokes a certain class) or availability (how easily similar cases come to mind). While the authors explicitly state that “in general, these heuristics are quite useful”, the focus is on the biases associated with such intuitive short-cuts e.g. insensitivity to base rates, sample size, and predictability. The deficiencies of heuristics are demonstrated using the “rational” judgement or choice as a comparator. However, while this is straight forward for situations in which the optimal solution is known, in most practically relevant situations the optimal solution is unknown or even unknowable. Therefore, Gigerenzer and colleagues modified this view by defining heuristics as efficient judgemental operations that deliberately use only part of the potentially available information and simple computation [9, 41]. Emphasis is put on exploiting “less-is-more” effects and on formalizing and evaluating heuristics (e.g. for use in decision support tools [43, 44]). This integrates heuristics coequally into the larger toolset of forecasting and decision support.

Regarding fit with the sport ecology, simple models (heuristics) are generally favoured by sparse data. Moreover, the corporeality of exercise offers the option to leverage the innate capacities of the human mind (e.g. perceiving physical exertion or recognizing movement patterns that indicate it in others) and thereby favors the validity of subjective cues. Finally, formalizing experiential knowledge as heuristics offers a potential hub between experiential knowledge and scientific evidence [43, 44]. Taken together, the general fit between heuristics and the sports ecosystem seems to be almost exemplary. Readers interested in the rationales and rules for selecting specific heuristics are referred to Gigerenzer et al. [45].

### Taming and Harnessing Subjective Judgements (Crowd Intelligence)

Despite the positive perspective on experiential knowledge and expert judgment presented above, there are also major downsides of this “human-driven” approach. These include limitations in attention, time and memory as well as the numerous biases introduced e.g. by limited or irrelevant information and wishful thinking [42]. Moreover, the informal process and verbal or even implicit judgements (as opposed to quantitative probability estimates) complicate the evaluation of performance. Taken together, subjective judgements are a serious option when trying to make forecasts in complex situations with incomplete information and very few specific precedents e.g. when trying to mitigate injury risk in an elite athlete. However, the risk of bias and a lack of (objective) verification of performance are downsides of this uniquely human contribution.

In fields that regularly deal with this conundrum when the stakes are high (e.g. intelligence analysis [46, 47]), techniques for mitigating these limitations have been developed. First and foremost, objective evaluation of subjective judgements is enabled by unambiguous targets (including criterion and timeline) and quantitative estimates [31, 46]. Of course, this requires a commitment to accountability, feedback and continuous improvement on the part of the raters. As a next step, the accuracy of individual raters may be increased by feedback and advice on good judgement practice (e.g. incremental updating of reference class information [48]). Beyond these basic measures, further improvements are mainly achieved by having not one but many raters [12, 46]—in exact analogy to averaging several measurements e.g. of VO_2max_ [49] to reduce the impact of measurement error. The concept of “crowd intelligence” or “wisdom of the crowd” dates back to the beginning of the democratic era [10] and posits that in many situations aggregating subjective judgements from a large number of independent raters (on average) leads to a more accurate estimate than the judgement of a single rater [50]—even in the case of superior expertise, experience [10] and access to exclusive information [46]. While initially simple averaging was used [10], today more sophisticated methods for aggregation are available [12, 31]. Today, the increase in accuracy achievable with aggregating individual judgements is well confirmed theoretically [51] as well as empirically (for examples from sport, see [52, 53]). Moreover, the requirements on the side of the crowd (e.g. diversity and access to the circumstance) as well as regarding the collection and aggregation of judgements (e.g. incentive and appropriate aggregation method) are understood [12, 31, 51]. Implementing the collection, aggregation and presentation of subjective judgments in a web application or smartphone app can be the final step of taming subjective judgements and integrating them into the harnessed team of forecasting and decision support tools [54]. Taken together, aggregated subjective judgments are a promising option for improving decision support in high-level sports—specifically in “unique” situations in which statistical learning is doomed to fail, clearcut heuristics are not available and the popularity of a sport provides a large and motivated “crowd” (e.g. football).

### Integrating Diverse Sources of Information and Knowledge

In the sports ecosystem, each of the approaches to learning, forecasting and decision-making is associated with considerable limitations. Therefore, it seems promising to search for synergies and complementary combinations—in particular between data-driven and human-driven strategies but also between existing knowledge and new data. Mundane (yet essential) examples are the support of experiential learning by formal training [45] (e.g. for obtaining a coaching licence) and common-sense-based plausibility checks. While there are countless ways to formally combine sources of information and knowledge, it may be helpful to identify two main categories: (1) Building upon preexisting knowledge and (2) Single-stage integration of results from diverse strategies.

#### Leveraging Prior Knowledge—Baysian Updating, Shrinkage, and Causal Inference

When the small number of precedents and / or the acceptability of extensive study requirements are limiting factors (as is typically the case in sport), prior knowledge—which may concern the magnitude and/or the causal structure of the effect in question—may be used to augment current data. Importantly, the usefulness of information from a larger reference class for decision making on the individual level is not a matter of course. Rather, generalizability from the group level to the individual case is gradually dependent on inter- and intraindividual variability in the outcome of interest [7] and / or the underlying structure of explanatory variables [55]. If interindividual variation is negligible (in other words, if the ergodicity-assumption holds), group-based information is directly applicable on the individual level. By contrast, if interindividual variation is very large (“non-ergodicity” [56]), group-based information is not helpful for individual level decision making. Between these two extremes, if interindividual variation is substantial but not dominant, group-based information can be used as a valuable starting point that can be fine-tuned with limited amounts of individual-level data.

##### Anchor and Adapt

Fortunately, at least in high-performance sport, we usually have a defendable prior expectation about the direction and magnitude of the effect(-s) in question. Options to formally implement a “hub” with new data range from using base rates as anchors[57], over “Bayesian updating” of informative priors [58, 59] and shrinking individual forecasts towards the group average [60], and pre-trained models in machine learning [2, 3]. Ultimately, these methods balance individuality and robustness by including an “anchor” based on a larger reference class. The following references provide some worked examples of the integration of preexisting knowledge and current data in sport [2, 58, 59, 61].

##### Leveraging Causal Knowledge

Insights into the causal structure of the effect under investigation can be used to gain more information from available data, specifically for improving out-of-sample predictive accuracy, robustness and interpretability [62]. While fully implementing causal inference arguably requires expert collaboration, explicitly specifying subject matter knowledge (or assumptions) in a causal diagram is already an important step enabling transparent scrutiny and identification of pitfalls [62]. It should be noted that existing causal knowledge or respective assumptions are involved in any trial even if the statistical analysis is purely data-driven and model-free (e.g. for selecting proper stratification criteria and standardization measures).

#### Integrating Diverse Perspectives—Dragonfly Eye Strategies and Triangulation

Supplementary knowledge and different perspectives may also arise in parallel, e.g. by applying several of the above strategies to the same use case. As already noted, integrating judgements from diverse and independent raters can reliably improve accuracy and avoid extreme outliers. This principle of “crowd intelligence” also applies to non-human sources. A salient example is ensemble methods in machine learning such as random forests (which rely on purposefully increased diversity and independence followed by aggregation). The combination of forecasts across different machine learning methods is referred to as “consensus forecasting” or “committee machine”. Of course, different, potentially complementary access routes may also be combined in a non-algorithmic way to gain a human-driven, composite assessment (dragonfly eye strategy) or to identify spurious extremes that may arise as artefacts of a particular method (plausibility control). In the context of scientific research, increasing the robustness of insights by combining diverse lines of evidence is known as “triangulation” [63].

## Conclusion

Taken together, the above considerations call for (deliberate and reflective) street-smart decision-making in sport: waiving extensive proceedings or authorities that are axiomatically considered optimal (even if they would theoretically be under ideal conditions) for strictly outcome-oriented approaches that are less elegant and ambitious but adapted to and proven in the environment in question. Specifically, in the sport ecosystem, key amendments to data-driven evidence and individual eminence are:

- Using the head start provided by *preexisting knowledge*
- Targeting the sweet spot of model complexity by *deliberate simplification*
- Harnessing uniquely *human contributions*
- Replacing gold-standard dogmas by using *synergies* between diverse approaches

## Data Availability

Not applicable.
